# Cerebrovascular complications and outcomes of critically ill adult patients with infective endocarditis

**DOI:** 10.1186/s13613-022-01086-6

**Published:** 2022-12-30

**Authors:** Thomas Rambaud, Etienne de Montmollin, Pierre Jaquet, Augustin Gaudemer, Eric Mariotte, Sonia Abid, Marylou Para, Claire Cimadevilla, Bernard Iung, Xavier Duval, Michel Wolff, Lila Bouadma, Jean-François Timsit, Romain Sonneville

**Affiliations:** 1 Université Paris-Cité, INSERM UMR1148, Team 6, 75018 Paris, France; 2 Department of Intensive Care Medicine, AP-HP. Nord, Hôpital Bichat - Claude Bernard, Paris, France; 3 Département de Réanimation Médico-Chirurgicale, APHP Hôpital Avicenne, Bobigny, France; 4 Université Paris Cité, INSERM UMR1137, IAME, 75018 Paris, France; 5 Department of Radiology, AP-HP, Hôpital Bichat-Claude Bernard, 75018 Paris, France; 6 Department of Intensive Care Medicine, AP-HP, Hôpital Saint-Louis, 75010 Paris, France; 7 Surgical Intensive Care Unit, Saint Louis Hospital, AP-HP, Paris, France; 8 Department of Cardiac Surgery, AP-HP, Hôpital Bichat - Claude Bernard, 75018 Paris, France; 9 Department of Cardiology, AP-HP, Hôpital Bichat - Claude Bernard, 75018 Paris, France; 10 Université Paris-Cité , INSERM UMR1148, Paris, France; 11 Department of Infectious Diseases, AP-HP, Hôpital Bichat-Claude Bernard, 75018 Paris, France; 12 GHU Paris Psychiatrie & Neurosciences, Paris, France

**Keywords:** Endocarditis, Stroke, Thoracic surgery, Neuro-critical care

## Abstract

**Background:**

Neurological complications are associated with poor outcome in patients with infective endocarditis (IE). Although guidelines recommend systematic brain imaging in the evaluation of IE patients, the association between early brain imaging findings and outcomes has never been evaluated in critically ill patients. We aimed to assess the association of CT-defined neurological complications with functional outcomes of critically ill IE patients.

**Methods:**

This retrospective cohort study included consecutive patients with severe, left-sided IE hospitalized in the medical ICU of a tertiary care hospital. Patients with no baseline brain CT were excluded. Baseline CT-scans were classified in five mutually exclusive categories (normal, moderate-to-severe ischemic stroke, minor ischemic stroke, intracranial hemorrhage, other abnormal CT). The primary endpoint was 1-year favorable outcome, defined by a modified Rankin Scale score of 0–3.

**Results:**

Between 06/01/2011 and 07/31/2018, 156 patients were included. Among them, 87/156 (56%) had a CT-defined neurological complication, including moderate-to-severe ischemic stroke (*n* = 33/156, 21%), intracranial hemorrhage (*n* = 24/156, 15%), minor ischemic stroke (*n* = 29/156, 19%), other (*n* = 3/156, 2%). At one year, 69 (45%) patients had a favorable outcome. Factors negatively associated with favorable outcome in multivariable analysis were moderate-to-severe ischemic stroke (OR 0.37, 95%CI 0.14 − 0.95) and age (OR 0.94, 95%CI 0.91–0.97). By contrast, the score on the Glasgow Coma Scale was positively associated with favorable outcome (per 1-point increment, OR 1.23, 95%CI 1.08–1.42). Sensitivity analyses conducted in operated patients revealed similar findings. Compared to normal CT, only moderate-to-severe ischemic stroke was associated with more frequent post-operative neurological complications (*n* = 8/23 (35%) vs *n* = 1/46 (2%), *p* < 0.01).

**Conclusion:**

Moderate-to-severe ischemic stroke had an independent negative impact on 1-year functional outcome in critically ill IE patients; whereas other complications, including intracranial hemorrhage, had no such impact.

**Supplementary Information:**

The online version contains supplementary material available at 10.1186/s13613-022-01086-6.

## Introduction

Despite progress in its management, infective endocarditis (IE) is still associated with high in-hospital and long-term mortalities [1, 2]. Neurological complications occur in 20–55% of patients [3–5] with left-sided IE, and have been associated with increased mortality [1, 6–9] and poorer functional outcome [4]. Most IE patients with a neurological complication have a surgical indication, but whether surgery should be contraindicated or delayed in such a situation is still debated [10]. Consistently, international guidelines recommend use of cerebral imaging during pre-operative evaluation in acute IE patients with neurological symptoms [11].

IE patients frequently require intensive care unit (ICU) admission [9] and critically ill IE patients harbor specific features such as greater involvement of *Staphylococcus aureus*, higher rates of neurological complications, and increased long-term mortality [12, 13]. In critically ill patients, brain imaging is paramount as confounding factors such as septic or metabolic encephalopathy as well as use of sedative drugs may impact clinical neurological evaluation. Thus, it is unsure whether results from studies conducted in less severe patients and using a symptom-based classification of neurological complications can be extrapolated to critically ill patients. However, few studies investigated neurological complications in critically ill IE patients and results from these studies are contradictory, especially regarding the impact of these complications on prognosis. While most retrospective studies were not able to detect an association of neurological complications with outcomes [7, 14], a prospective multicenter study identified an independent association between neurological failure (Glasgow Coma Scale (GCS) score < 10) [5] and mortality. Furthermore, a recent retrospective multicenter study identified an association between stroke (either ischemic or hemorrhagic) and mortality [9]**.** In addition to differences in population and possible referral bias, these conflicting results probably reflect discrepancies in definitions and diagnosis of neurological complications [5, 7, 9, 14]. Specifically, none of these studies reported a systematic use of brain imaging (computed tomography (CT) scan or magnetic resonance imaging (MRI)). Although MRI has been proposed for the initial evaluation of IE patients, this imaging technique is impractical for the unstable critically ill patient [15] and CT-scan remains the neuroimaging technique of choice [5]**.** However, to the best of our knowledge, no specific study has evaluated the diagnostic and prognostic values of systematic CT-scan imaging in critically ill patients with IE.

Our aim was to assess the association of CT-defined baseline neurological complications with functional outcomes in critically ill patients with left-sided IE.

## Methods

### Design

We conducted, between 06/01/2011 and 07/31/2018, a single center retrospective cohort study on consecutive IE patients admitted to the medical ICU of a tertiary referral center for cardiac surgery and IE treatment, in Paris, France. In this center, patients with IE are treated by a multidisciplinary IE team including cardiologists, infectious disease specialists, microbiologists, cardiac surgeons, neurologists, radiologists, anesthesiologists and intensive care specialists.

### Patients

Patients were screened via a computer search in the local discharge database according to the ICD-10 (code I33 (acute and subacute endocarditis) and/or I38 (endocarditis, valve unspecified)). We included all consecutive patients ≥ 18 years old diagnosed with a definite, active, severe, left-sided IE requiring ICU admission. Definite IE was defined according to the modified Duke criteria [11]. IE was defined as active if the patient was admitted to the ICU before or within the first 30 days of antimicrobial treatment. Severe IE was defined as a SOFA score ≥ 2, indicating the presence of an organ dysfunction [16]. Left-sided IE was defined as IE involving mitral and/or aortic valves. Cases of both left- and right-sided IE or with left-sided IE associated with infection of a cardiac implantable electronic device (CIED) were also considered as left-sided IE. Exclusion criteria were: (1) patients transferred to the ICU after cardiac surgery for IE, (2) ICU-acquired IE, (3) patients already included in the study for a previous IE and (4) patients with no available baseline brain CT. Baseline brain CT was defined as the first brain CT performed after ICU admission or, if no brain CT had been done in the ICU, as the last brain CT before ICU admission. CT-scans performed after IE surgical treatment or > 10 days before ICU admission were not considered as baseline brain CT.

### Initial workup

All IE patients received an initial standardized diagnostic workup including clinical evaluation, echocardiography (trans-thoracic echocardiography (TTE) and trans-esophageal echocardiography (TOE)), routine biological workup and a bacteriological workup including at least two blood sample cultures. All TTE/TOE were conducted by a senior cardiologist from the cardiac surgery department. The vegetation length was measured in various planes during the first echocardiogram and on follow-up when available, and the maximal length was used for analysis. Other explorations including coronary angiography, CT scan of thorax, abdomen and pelvis (CT-TAP) and brain vascular imaging were performed when deemed necessary.

### Ethics

The local ethics committee (Comité local d’éthique HUPSSD-Avicenne) approved the study (agreement CLEA-2021-191).

### Data collection

Each patient’s condition at ICU admission was assessed using the Sequential Organ Failure Assessment (SOFA) [16], and the GCS scores [17], both prospectively calculated. If the patient was sedated at ICU admission, the last GCS available before sedation was recorded and used in the analysis. If the initial brain-CT scan revealed a stroke, the clinical severity was assessed using the National Institute of Health Stroke Scale [18] (NIHSS). When necessary, the NIHSS was assessed retrospectively by a neurologist (TR) using a validated approach [19]. Neurological symptoms were assessed retrospectively by a neurologist (TR), based on the systematic initial clinical examination conducted by a senior intensivist. Immunodepression was defined as human immunodeficiency virus infection, malignancy, long-term use of corticosteroids or other immunosuppressants.

### IE

Community-acquired IE was defined as IE diagnosed within 48 h of hospital admission, for a patient who did not satisfy the criteria for health care-associated infection. Healthcare-associated IE was classified as nosocomial or non-nosocomial in accordance with current definitions [20]. IE diagnosis date was defined as the day on which the first positive blood culture was obtained or, in case of blood culture-negative IE, as the date of the first echocardiography revealing findings consistent with IE.

### Surgery

Indication for surgery was assessed throughout the ICU stay according to European guidelines [11] and further classified as emergency (within 24 h), urgent (within 7 days) or non-urgent (surgery can be postponed to allow 1 or 2 weeks of antimicrobial therapy).

### Neuroimaging

The first report of the baseline brain CT scan, carried out by a senior radiologist, was collected. Images from this CT-scan were re-analyzed by a neurologist (TR), using a standardized method and blinded to the first report of the imaging, clinical data, outcome and any other brain imaging undergone by the patient. Findings from the radiology report were then compared to the second interpretation and, in case of discrepancies, a third interpretation was made by another neurologist (PJ), also blinded to all relevant data and to both previous interpretations.

The presence of brain lesions was assessed on the baseline brain CT scan, according to an a priori defined grid: ischemic stroke (IS) including topography and volume (evaluated using the semi-quantitative ASPECT and PC-ASPECT scales [21, 22]), hemorrhagic transformation (HT) (classified according the ECASS-2 classification [23]), intraparenchymal hemorrhage (IPH), subarachnoid hemorrhage (SAH), subdural hemorrhage (SDH), extradural hemorrhage (EDH), abscess, empyema and mycotic aneurysm.

Baseline brain CT-scans were classified in five mutually exclusive categories: normal, moderate-to-severe ischemic stroke, minor ischemic stroke, cerebral hemorrhage and other infectious complications.

Moderate-to-severe ischemic stroke was defined (adapted from [6]) as an ischemic stroke involving the middle cerebral artery (MCA) territory with an ASPECT score ≤ 7 and/or involving the posterior cerebral artery territory with a PC-ASPECT score ≤ 7 and/or a multiterritorial ischemic stroke with at least two lesions ≥ 15 mm involving at least two distinct vascular territories. All ischemic strokes not meeting these criteria were classified as “minor ischemic stroke”. Cerebral hemorrhages included IPH, SAH, HT (except minor non-confluent petechiae [23]), SDH and EDH. Other infectious complications included cerebral abscess, empyema and diffuse cerebral edema (adapted from [6]).

Patients with several different radiological lesions were classified as follows: (1) patients with a normal baseline CT were classified in the “normal CT” category; (2) patients with moderate-to-severe ischemic stroke and any other radiological lesions were classified in the “moderate-to-severe ischemic stroke” category; (3) patients with a cerebral hemorrhage and either a minor ischemic stroke or another infectious complication were classified in the “cerebral hemorrhage” category; (4) patients with a minor ischemic stroke and another infectious complication were classified in the “minor ischemic stroke” category (Additional file 5: Figure S1 and Additional file 6: Figure S2).

### Endpoints

The primary endpoint was the proportion of patients with a favorable functional outcome, 1 year after ICU admission. Outcome was assessed using the modified Rankin Scale (mRS, ranging from 0 to 6 with a score of 0 indicating no disability, higher scores indicating more severe disability and 6 indicating death [24]). Favorable functional outcome was defined as a score on the mRS between 0 (asymptomatic) and 3 (moderate disability). The mRS was assessed using a standardized validated questionnaire [25], by reviewing the medical chart or, when the available data were insufficient, by contacting the patient’s treating physician. Secondary endpoint was the risk of severe post-operative neurological complications (defined as a new symptomatic neurological event with a new abnormality on any brain imaging and/or a new hospitalization or ICU admission due to a neurological event and/or death due to a neurological event) within 90 days after surgery.

Causes of death were classified as “neurological”, “heart failure or cardiogenic shock”, “sepsis or septic shock” or “other” by two neurointensivists (TR, RS) through analysis of medical charts.

Due to an expected very small number, patients with a baseline CT-scan classified as “other infectious complications” were excluded from outcome analysis.

### Statistical analysis

Data are expressed as counts and frequencies for categorical variables and medians [interquartile range] for quantitative variables. Univariate comparisons between subgroups were performed using χ^2^ test or Fisher exact test for categorical variables and Mann–Whitney test for continuous variables.

To identify independent predictors of unfavorable outcome at one year, we used a multivariable logistic regression model using a backward selection procedure among the following variables: baseline brain CT categories and all baseline characteristics associated with the outcome of interest in univariate analysis (*p* < 0.1, Additional file 1: Table S1). To avoid collinearity, when both a score including several components (such as the Charlson comorbidity index or the non-neurological SOFA) and one of its individual components were associated with the outcome of interest, only the multi-component score was included in the multivariable analysis. Log linearity of continuous variables was tested and, if necessary, variables were categorized according to clinically relevant cutoffs or at median values. Collinearity between variables and 2-by-2 interactions were tested. A separate regression analysis was performed for each category of baseline brain CT. The magnitude of association with the outcome was expressed as an odds ratio (OR) and 95% confidence intervals (95% CI). Missing data were handled with pairwise deletion method. All tests were two-sided, and *p*-value < 0.05 was considered statistically significant. All analyses were performed using R, Version 3.5.2 (R Project for Statistical Computing, https://www.r-project.org).

## Results

### Population

**Fig. 1:**
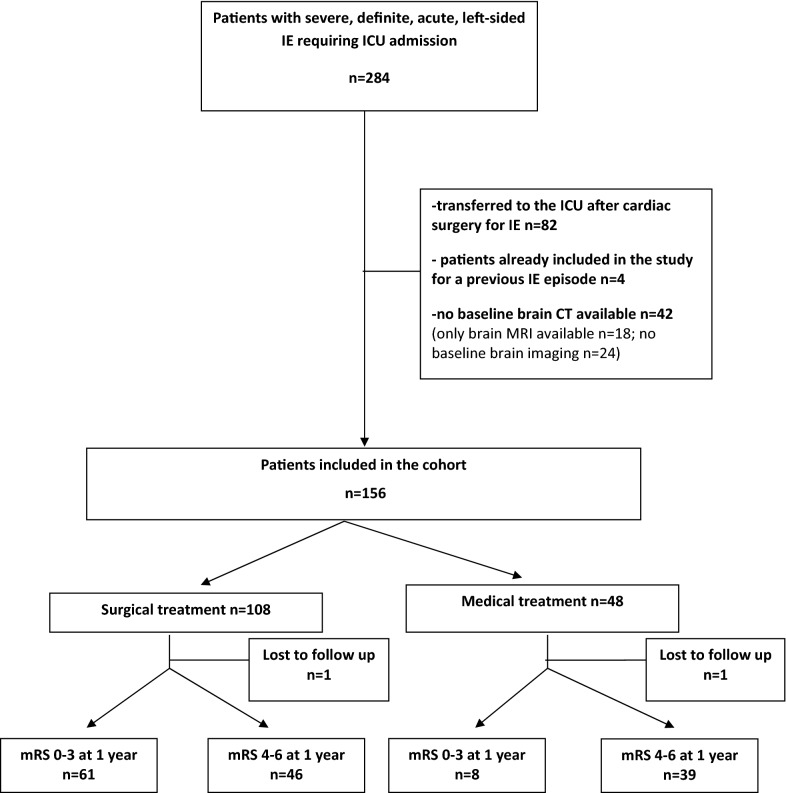
Flowchart of the cohort

A total of 284 patients fulfilled the inclusion criteria during the study period, of which 128 were excluded due to pre-specified exclusion criteria (Fig. 1). 156 patients (age 63 years old (54–70), 71% male, Table 1) were included in the cohort. There was no significant difference in demographic characteristics and outcomes between the 42 patients excluded because of absence of baseline brain CT and those included in the cohort (Additional file 1: Table S1). 150/156 patients (96%) had no disability (mRS < 2) before the index episode. The time between IE diagnosis and ICU admission was 2 (1–7) days. Baseline contrast brain CT was performed in 132/156 (85%) patients and non-contrast CT in the remaining 24/156 (15%). The time between IE diagnosis and baseline brain CT was 3 (1–8) days. A TTE was carried out in 156/156 (100%) patients, a TOE in 145/156 (93%) and a CT-TAP in 127/156 (81%).

**Table 1:** Baseline characteristics

Characteristics	Total (*n* = 156)	Normal CT (*n* = 67)	Moderate-to-severe ischemic stroke (*n* = 33)	Minor ischemic stroke (*n* = 29)	Cerebral hemorrhage (*n* = 24)	p (4 group comparison)
Demography						
Age (years)	63 [54–70]	63 [55–72]	62 [53–68]	68 [59–72]	63 [51–66]	0.06
Male sex	110 (71%)	45 (67%)	25 (76%)	20 (69%)	17 (71%)	0.85
Pre-existing conditions						
Charlson score	1 [0–3]	2 [0–4]	0 [0–1]	2 [1–4]	0 [0–2]	< 0.001
Diabetes	46 (30%)	22 (33%)	8 (24%)	13 (45%)	3 (13%)	0.06
Chronic kidney disease (a)	30 (19%)	15 (22%)	3 (9%)	5 (21%)	7 (24%)	0.38
Anticoagulant treatment	48 (31%)	19 (28%)	11 (23%)	10 (35%)	8 (33%)	0.9
Antiplatelet treatment	34 (22%)	20 (30%)	6 (18%)	7 (24%)	1 (4%)	0.06
CIED	14 (9%)	8 (12%)	3 (9%)	0 (0%)	3 (13%)	0.28
Valvular predisposition	66 (42%)	27 (40%)	16 (48%)	11 (38%)	12 (50%)	0.7
Prosthetic valve	44 (28%)	20 (30%)	11 (33%)	8 (28%)	5 (21%)	0.77
Other valvular disease	22 (14%)	7 (10%)	5 (15%)	3 (10%)	7 (29%)	0.14
IE characteristics						
*Staphylococcus aureus*	84 (54%)	35 (52%)	23 (70%)	9 (31%)	17 (71%)	0.007
*Enterococcus* sp	15 (10%)	7 (10%)	2 (6%)	6 (21%)	0 (%)	0.07
Other Streptococci	34 (22%)	15 (22%)	5 (15%)	10 (34%)	3 (13%)	0.18
Blood culture negative	6 (4%)	1 (1%)	2 (6%)	2 (7%)	1 (4%)	0.5
Prosthetic valve IE	38 (24%)	17 (25%)	10 (30%)	6 (21%)	5 (21%)	0.80
IE topography						
Mitral	75 (48%)	33 (49%)	13 (39%)	13 (46%)	15 (63%)	0.39
Aortic	61 (39%)	25 (37%)	18 (55%)	11 (39%)	6 (25%)	0.15
Both	20 (13%)	9 (13%)	2 (6%)	4 (14%)	3 (13%)	0.71
Vegetation > 15 mm	69 (44%)	21 (31%)	16 (49%)	17 (59%)	13 (54%)	0.04
Severe regurgitation	64 (41%)	27 (40%)	8 (24%)	15 (52%)	12 (50%)	0.11
Cardiac abscess or fistula	42 (27%)	13 (19%)	12 (36%)	8 (28%)	7 (29%)	0.32
Extra-neurological CT-defined embolism	70 (55%)	30 (45%)	18 (55%)	15 (52%)	11 (46%)	0.79
Baseline clinical characteristics						
SOFA	7 [4–10]	6 [4–10]	7 [4–11]	7 [4–10]	5 [5–11]	0.97
“non-neurological” SOFA	6 [3–9]	6 [4–8]	6 [3–9]	7 [3–9]	6 [4–9]	0.9
Mechanical ventilation	89 (57%)	31 (46%)	20 (61%)	23 (79%)	12 (50%)	0.02
IV catecholamines	65 (42%)	24 (36%)	15 (45%)	10 (35%)	9 (38%)	0.80
Lactate > 2 mmol/L	40 (26%)	21 (31%)	6 (18%)	7 (24%)	6 (25%)	0.51
GCS	14 [12–15]	15 [14, 15]	14 [12–15]	14 [13–15]	13 [11–14]	0.81
GCS < 10	30 (19%)	12 (18%)	8 (24%)	3 (10%)	5 (21%)	0.54

a. eGFR < 60 mL.min^−1^.1.73 m^−2^ for > 3 months^−1^ detailed characteristics of the 3 patients with “other abnormal CT” are not shown here due to small number

1. Chen TK, Knicely DH, Grams ME. Chronic Kidney Disease Diagnosis and Management. JAMA. 2019;322:1294–1304

*IE* infective endocarditis, *IV* intra-venous, *CIED* cardiac implantable electronic device, *LVEF* left ventricular ejection fraction, *CT* computed tomography, *SOFA* Sequential Organ Failure Assessment, *GCS* Glasgow Coma Scale, *ICU* intensive care unit

### Neurological complications

87/156 (56%) patients had ≥ 1 neurological complication on baseline brain CT scan. Detailed results of baseline brain CT scan are shown in Table 2. Using the previously described classification rule, patients were classified as follows: normal brain CT scan (*n* = 67, 43%), moderate-to-severe ischemic stroke (*n* = 33, 21%), minor ischemic stroke (*n* = 29, 19%), cerebral hemorrhage (*n* = 24, 15%), other infectious complications (*n* = 3, 2%). Among patients with ≥ 1 lesion on baseline CT scan, 41/87 (46%) presented with focal neurological symptoms, 29/87 (33%) with confusion, 18/87 (20%) with a decreased level of consciousness, and 20/87 (22%) had no neurological symptoms. Baseline NIHSS was 10 (3–18) in patients with moderate-to-severe IS, 2 (0–6) in patients with minor ischemic stroke and 5 (2–14) in patients with cerebral hemorrhage. The ASPECT score was 9 (8, 9) in the 52 patients with an MCA ischemic stroke and the pc-ASPECT score was 9 (8, 9) in the 24 patients with a posterior circulation IS. 2/75 (3%) patients with ischemic stroke received and intravenous thrombolysis and 1/75 (2%) an endovascular recanalization treatment. 15/156 (9%) patients underwent a catheter angiography, and a mycotic aneurysm was identified in 4 of these patients. All 4 mycotic aneurysms identified received endovascular treatment. No patient underwent a neurosurgical procedure.

**Table 2:** Details of CT-defined neurological complications identified on baseline brain CT

Type of complication	Number of lesions in the whole cohort (*n* = 156)	%
Ischemic complications (≥ 1)	75/156	48
Small (≤ 15 mm) ischemic stroke only	21/75	28 (a)
Multiterritorial ischemic stroke	35/75	47 (a)
Anterior circulation ischemic stroke only	41/75	55 (a)
Posterior circulation ischemic stroke only	8/75	11 (a)
Anterior and posterior circulations	24/75	32 (a)
Mass effect	5/75	7 (a)
Hemorrhagic complications (≥ 1)	36/156	24
Intraparenchymal hemorrhage (c)	17/36	47 (b)
Subarachnoid hemorrhage	19/36	53 (b)
Subdural hematoma	2/36	6 (b)
Hemorrhagic transformation of an ischemic stroke	13/36	44 (b)
Other infectious complications		
Mycotic aneurysm (d)	4/156	3
Abscess/empyema	4/156	3

*CT* computed tomography

(a). % among *n* = 75 patients with ischemic lesions

(b). % among *n* = 36 patients with hemorrhagic lesions

(c). Including *n* = 8 with significant mass effect

(d). *n* = 4/4 confirmed by catheter angiography, all 4 patients received endovascular aneurysm treatment

### Surgical treatment

Emergency or urgent surgery was indicated in 136/156 (87%) patients and surgery was performed in 108/136 (79%) of them. 28/136 (21%) patients were denied surgery despite emergency or urgent indication due to (non-exclusive reasons): neurological complications (14/28, 50%), co-existing medical condition(s) (14/28, 50%), multi-organ failure (8/28, 29%), expected surgical technical difficulties (4/28, 14%), improvement with medical treatment (2/28, 8%) or patient choice (1/28, 4%). The proportion of patients denied surgery despite indication did not differ significantly between patients with or without CT-defined neurological complications. The delay between IE diagnosis and surgery was 7 (4–15) days (moderate-to-severe ischemic stroke 9 (4–14) days, minor ischemic stroke 5 (4–12) days, cerebral hemorrhage 10 (4–19) days, and normal brain CT 6 (3–16) days, *p* = 0.71).

### One-year outcomes

**Fig. 2:**
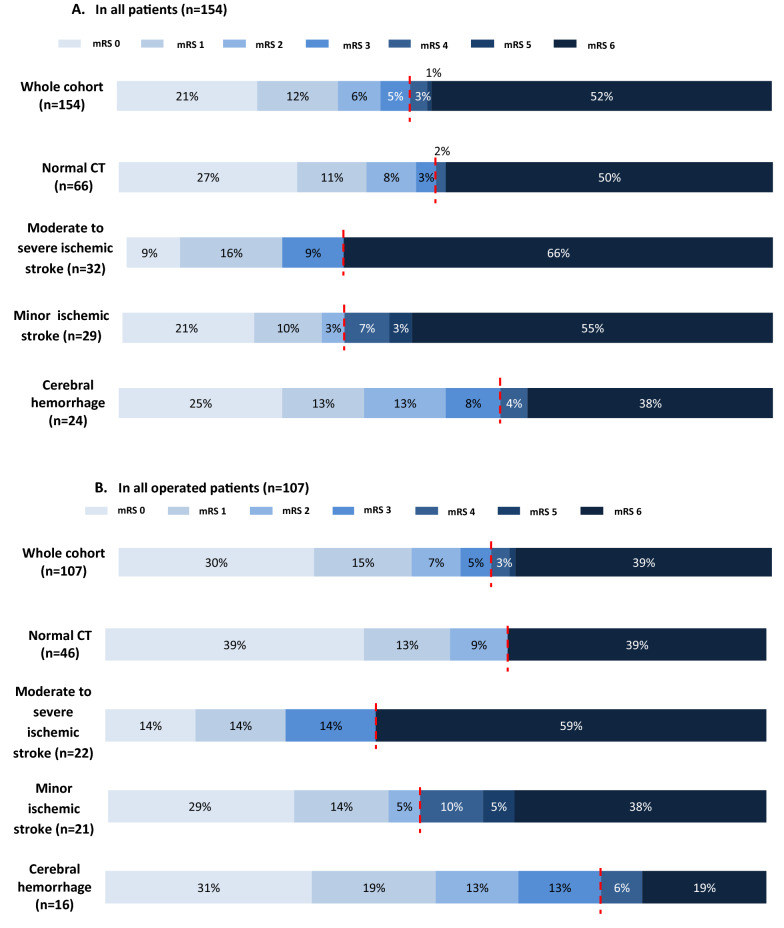
Rankin score distribution at one year: **A**. Rankin distribution at one year in the whole cohort, according to results of baseline CT-scan; **B**. Rankin distribution at one year in operated patients according to results of baseline pre-operative CT-scan. In both A and B, the red line indicates the limit between patients with favorable (mRS 0–3) or unfavorable (mRS 4–6) outcome. *CT* computed tomography

One year after IE diagnosis, 2/156 (1%) patients were lost to follow-up and mRS was available for 154 patients. 69/154 (45%) patients had a favorable outcome (mRS 0–3) at one year (Fig. 2). Among patients alive at one year, only 5/74 (7%) were severely disabled (mRS 4–5). In addition to the baseline brain CT scan category, variables associated with outcome in univariate analysis (Additional file 2: Table S2) included in the multivariate model were age, baseline GCS score, non-neurological SOFA, Charlson comorbidity index and involvement of the mitral valve. In multivariate analysis, moderate-to-severe ischemic stroke was independently associated with a lower probability of favorable functional outcome at one year (OR for achieving favorable outcome = 0.37 [IC 95% 0.14–0.95], *p* = 0.038) (Table 3). In contrast, cerebral hemorrhage, minor ischemic stroke or a normal CT-scan were not independently associated with one-year outcome (Additional file 3: Table S3). A sensitivity analysis including the variable "severe regurgitation" in the multivariable model yielded similar results (Additional file 4: Table S4). At 1 year, 80/154 (52%) patients had died. Causes of death were neurological in 26/80 patients (33%), sepsis or septic shock in 36/80 (45%), heart failure or cardiogenic shock in 11/80 (14%) and other in 7 (9%) (Table 4). Most deaths due to neurological causes (22/26, 85%) occurred after withdrawal of life-sustaining therapies. Median delay between IE diagnosis and death was 22 (9–43) days.

**Table 3:** Multivariable analysis of factors associated with favorable functional outcome (mRS 0–3) at one year

Variable	Univariate OR [95% CI]	Univariate p	Multivariate OR [95% CI]	Multivariate *p*
A. All patients (*n* = 154)				
CT-defined moderate-to-severe ischemic stroke	0.7 [0.3–1.5]	0.34	**0.37 [0.14–0.95]**	**0.038**
Age	0.9 [0.9–1.0]	< 0.001	**0.94 [0.91–0.97]**	** < 0.001**
GCS score	1.2 [1.1–1.4]	< 0.001	**1.23 [1.08–1.42]**	**0.003**
Charlson index ≥ 2	0.6 [0.3–1.1]	0.085	0.54 [0.25–1.18]	0.12
Non-neurological SOFA ≥ 5	0.5 [0.2–0.9]	0.021	–	–
Mitral valve involvement	0.5 [0.3–1.0]	0.05	0.58 [0.28–1.20]	0.14
B. Operated patients (*n* = 107)				
CT-defined moderate-to-severe ischemic stroke	0.4 [0.2–1.1]	0.09	**0.30 [0.01–0.93]**	**0.037**
Age	0.9 [0.9–1.0]	0.009	**0.96 [0.93–0.99]**	**0.018**
GCS score	1.2 [1.1–1.4]	0.007	**1.24 [1.05–1.47]**	**0.012**
Charlson index ≥ 2	0.4 [0.2–1.0]	0.05	**0.36 [0.14–0.90]**	**0.03**
Non-neurological SOFA ≥ 5	0.5 [0.3–1.2]	0.13	–	–
Mitral valve involvement	0.6 [0.3–1.3]	0.2	–	–

A separate regression analysis was performed for each baseline brain CT category. Minor ischemic stroke, cerebral hemorrhage and normal CT were not significantly associated with 1-year favorable outcome neither in the principal analysis, nor in the operated population. Mitral valve involvement, non-neurological SOFA ≥ 5 and charlson comorbidity index (whole population only) were not retained for the final model during the backward selection procedure (*p* > 0.2). Variables significantly associated with outcome in multivariable analysis (*p* < 0.05) are displayed in bold

*CT* computed tomography, *GCS* Glasgow Coma Scale, *SOFA* Sequential Organ Failure Assessment

**Table 4:** Surgery and survival according to baseline CT scan categories

Characteristics	Total (*n* = 156)	normal CT (*n* = 67)	Moderate-to-severe ischemic stroke (*n* = 33)	Minor ischemic stroke (*n* = 29)		Cerebral hemorrhage (*n* = 24)	*p* (4 group comparison)
Emergency/urgent surgical indication	136 (87%)	56 (83%)	29 (88%)		27 (93%)	21 (88%)	0.67
Patients operated	108 (79%)	46 (69%)	23 (70%)		21 (72%)	16 (67%)	0.98
ICU mortality	64 (41%)	25 (37%)	17 (51%)		14 (48%)	7 (29%)	0.4
1-year mortality	80 (51%)	33 (49%)	21 (64%)		16 (55%)	9 (38%)	0.3
Including neurological causes	26/80 (33%)	3/33 (9%)	13/21 (62%)		4/16 (25%)	5/9 (56%)	< 0.01
Including sepsis or septic shock	36/80 (45%)	23/33 (70%)	2/21 (10%)		8/16 (50%)	3/9 (33%)	< 0.01
Including heart failure	11/80 (14%)	4/33 (12%)	4/21 (19%)		2/16 (13%)	1/9 (11%)	0.9

Data concerning the 3 patients with “other abnormal CT” are not shown here due to small number

### One-year outcomes in operated patients

Among 108 patients who underwent emergency or urgent surgery, one was lost to follow-up and 61/107 (57%) had a favorable outcome (mRS 0–3) at one year. (Fig. 2). In multivariate analysis, moderate-to-severe IS, age and GCS were independently associated with unfavorable functional outcome in operated patients (Table 3). At one year, 42/108 (39%) operated patients had died. A total of 12/108 (11%) operated patients had severe post-operative neurological complication within 90 days following surgery (Table 5). Compared to patients with normal brain CT scan, only patients diagnosed with a moderate-to-severe ischemic stroke had a significantly higher rate of post-operative severe neurological complications (8/23 (35%) vs 1/46 (2%), *p* < 0.01). Severe neurological complications were diagnosed 14 [IQR 5;21] days after surgery. Twenty-eight patients underwent surgery less than 4 weeks after IE diagnosis (median 10 [IQR 4;15] days) despite a theoretical neurological contraindication: severe ischemic stroke causing coma (*n* = 10) or cerebral hemorrhage (*n* = 18 including: HT *n* = 5/18; SAH *n* = 4/18; IPH *n* = 2/18; association of SAH and IPH *n* = 5/18 and SDH *n* = 2/18). Half of these patients (14/28) had a favorable outcome (mRS = 0–3) at one year. Patients operated despite hemorrhagic neurological contraindication achieved a favorable outcome more frequently than patients operated despite ischemic contraindication (mRS 0–3 at one year 67% vs 20%, *p* = 0.046). All but one patient operated despite a hemorrhagic contraindication underwent a contrast brain CT and catheter angiography was realized in all patients with an aneurysm identified on contrast CT (*n* = 3).

**Table 5:** Details of post-operative severe neurological complications

Type of complication	Moderate-to-severe ischemic stroke (23 operated patients)	Minor ischemic stroke (21 operated patients)	Cerebral hemorrhage (16 operated patients)	Normal baseline CT (46 operated patients)
Ischemic complications	4 (17%)	2 (10%)	1 (6%)	0 (0%)
New ischemic stroke in a previously unaffected territory	3 (13%)	2 (10%)	1 (6%)	0 (0%)
New ischemic stroke in an already affected territory	1 (4%)	0 (0%)	0 (0%)	–
Hemorrhagic complications	4 (17%)	0 (0%)	0 (0%)	1 (2%)
Hemorrhagic transformation	2 (9%)	0 (0%)	0 (0%)	–
New intraparenchymal hemorrhage	2 (9%)	0 (0%)	0 (0%)	1 (2%)
Enlargement of a pre-operative hemorrhage	0 (0%)	0 (0%)	0 (0%)	–

*CT* computed tomography

## Discussion

In this large retrospective cohort of critically ill IE patients requiring ICU admission, neurological complications identified on baseline CT scan were diagnosed in 56% of patients. We identified an independent association between moderate-to-severe ischemic stroke and poor long-term functional outcome. In contrast, outcomes of patients with a cerebral hemorrhage were not different to those of patients with a normal baseline brain CT scan.

Outcome was favorable in 45% patients and only a very limited number of patients (3%) survived with a severe disability (mRS 4 or 5).

Our multivariate model identified three independent prognostic factors at one year, namely baseline GCS, moderate-to-severe ischemic stroke and age. This result highlights the value of a multimodal neurological evaluation in these patients. We report here a standardized and reproducible classification, based on a double reading of systematic brain-CT. In line with results obtained in less severe patients [3, 6, 26], CT-defined moderate-to-severe ischemic stroke was the only neurological complication independently associated with functional outcome in our study.

Among patients with cerebral hemorrhage, we identified a wide variety of bleeding sites, likely reflecting the variety of underlying mechanisms. We report two SDH, corroborating a recent report of mycotic aneurysms complicated by SDH [27], a rare complication that clinicians should be aware of.

Among operated patients, 11% presented a post-operative severe neurological complication and only moderate-to-severe ischemic stroke was associated with an increased incidence of these complications. Neurological ischemic post-operative complications were more frequent than hemorrhagic ones in our population, an observation consistent with recent reports [28].

One-year functional outcome and the incidence of severe neurological post-operative complications were not different between patients with or without cerebral hemorrhage in our study. It is of special interest that no hemorrhagic neurological complications occurred in patients operated despite a cerebral hemorrhage. In our study, 26% of operated patients had a neurological contraindication according to current guidelines [11]. Among these patients, for whom only very limited data exist, functional outcome was significantly better in patients with hemorrhagic contraindications than in patients with ischemic contraindications. The impact of cerebral hemorrhage on outcome among operated patients has been evaluated by several studies, all observational and retrospective, with conflicting results. Some recent studies report a rate of post-operative neurological complications lower than previously described in these patients [6, 14, 29–32]. Most international guidelines recommend to delay surgery for ≥ 4 weeks in patients with cerebral hemorrhage [10, 11] but some authors suggest shortening this delay, especially in patients with urgent indications [29]. We report here a favorable outcome in 50% patients operated despite a theoretical neurological contraindication. Due to the retrospective nature of our study, all patients operated despite a neurological contraindication were selected patients, including many patients with a small or moderate cerebral hemorrhage and a contrast CT-scan ruling out any aneurysm. Our data, although retrospective and with a limited number of patients, question current guidelines and suggest that larger prospective studies are warranted.

Our study has limitations, including those inherent to its retrospective monocentric design. We chose to conduct a separate regression analysis for each baseline CT scan category, comparing for each CT scan result the patients with this specific result vs all the others in terms of outcome. This choice is arguable as a single regression analysis considering “baseline CT scan result” as a 4-category variable could also have been conducted. Our choice was based on the two following considerations: first, this methodology has been used previously in studies exploring prognostic factors of various diseases. Most importantly, the exact same methodology has been used in an important previous study exploring the impact of CT scan on outcome in 1345 non critically ill IE patients 6. Thus, this choice allows an easier comparison with already published data concerning less severe patients. Second, due to a relatively small number of events (69 one-year favorable outcome), the number of degrees of freedom of the multivariable analysis must be limited. Using a 4-category “CT scan result” variable would have increased this number. The exclusion of patients without available baseline CT scan, representing 14% of the patients screened for inclusion in our cohort, likely induced a misestimation of the incidence of neurological complications. This exclusion likely concerned both the most severe patients, which may have been considered too unstable to undergo brain imaging, and the less severe patients without neurological symptoms, in which the probability of a neurological lesion may have been estimated low. Of note, we observed no outcome differences between patients excluded due to absence of baseline CT scan and patients included in the cohort. Decision to perform a brain imaging is strongly encouraged in our center, as assessed by the high proportion of screened patients (> 90%) who underwent such procedures. Still, the presence of residual unmeasured confounders remains possible. The nature of the study center (a tertiary referral center) may also induce a bias as patients with complicated forms of IE and surgical indications may be over-represented in such a center. Finally, we report the use of systematic CT-scan and not of MRI, as previously reported in less severe patients. This may have led to underdiagnosis of neurological complications, especially small ischemic stroke and microbleeds. However, as neither small infarcts nor cerebral hemorrhage were associated with the outcomes in our study, it is unsure whether the use of systematic MRI would allow a better prognostication of critically ill patients.

## Conclusion

CT-defined neurological complications are frequent among critically ill patients with IE admitted to the ICU. Among these complications, only moderate-to-severe ischemic stroke was independently associated with unfavorable one-year functional outcome. Neurological post-operative complications were more frequent in patients with moderate-to-severe ischemic stroke compared to other patients, especially those with pre-operative cerebral hemorrhage. Our data suggest that in patients with severe IE, brain CT studies performed upon ICU admission provide valuable information for the diagnosis of neurologic complications. Moreover, the identification of moderate-to-severe ischemic stroke at the early phase of IE may have important prognostic implications.

## Supplementary Information

**Additional file 1: Table S1. **Comparison of patients excluded due to absence of baseline brain CT and patients included in the cohort.

**Additional file 2: Table S2. **Univariable analysis of baseline characteristics associated with outcome.

**Additional file 3: Table S3.** Multivariate analysis of factors associated with favorable outcome (mRS 0-3) in the whole cohort at one year for brain CT categories others than “moderate-to-severe ischemic stroke”.

**Additional file 4: Table S4.** Sensitivity analysis including the variable “severe regurgitation” of factors associated with favorable functional outcome (mRS 0-3) at one year.

**Additional file 5: Figure S1.** Details of baseline brain CT classification IS= Ischemic stroke HT=hemorrhagic transformation of an ischemic stroke

**Additional file 6: Figure S2.** Brain CT of four different patients from mentioned groups A. minor stroke of a distal branch of the left middle cerebral artery (ASPECTS 9/10, minor ischemic stroke group), B. large stroke involving the right middle cerebral artery with midline shift due to edema and mass effect (ASPECTS 3/10, moderate-to-severe ischemic stroke), C. intraparenchymal in left centrum semiovale with swirl sign (cerebral hemorrhage group) and D. hemorrhagic transformation of a left posterior cerebral artery stroke (also cerebral hemorrhage group).

## Data Availability

The datasets used and/or analyzed during the current study are available from the corresponding author on reasonable request.
