# Supplementary material for: Cerebrovascular complications and outcomes of critically ill adult patients with infective endocarditis
Source: Ann Intensive Care. 2022 Dec 30;12:119. doi: 10.1186/s13613-022-01086-6 (PMC9803797; doi:10.1186/s13613-022-01086-6)
Supplement: Supplementary file 1 — Additional file 1: Table S1. Comparison of patients excluded due to absence of baseline brain CT and patients included in the cohort. [file 13613_2022_1086_MOESM1_ESM.docx]

**Additional file Table S1: comparison of patients excluded due to absence of baseline brain CT and patients included in the cohort**

| **Characteristics** | **Patients with baseline brain-CT included in the cohort (n=156)** | **Patients excluded due to missing baseline brain CT (n=42)** | **p** |
| --- | --- | --- | --- |
| **Demography** |  |  |  |
| Age (years) | 63 [54-70] | 65 [56-76] | 0.1 |
| Male sex | 110 (71%) | 30 (71%) | 0.9 |
| **Pre-existing conditions** |  |  |  |
| Charlson score | 1 [0-3] | 2 [0-3] | 0.06 |
| Diabetes | 46 (30%) | 12 (29%) | 0.9 |
| Chronic kidney disease (a) | 30 (19%) | 6 (14%) | 0.4 |
| Immunosuppression | 27 (17%) | 13 (31%) | 0.08 |
| IV drug use | 7 (5%) | 2 (5%) | 0.9 |
| Stroke | 12 (8%) | 3 (7%) | 0.5 |
| Anticoagulant treatment | 48 (31%) | 8 (20%) | 0.2 |
| Antiplatelet treatment | 34 (22%) | 12 (30%) | 0.3 |
| CIED | 14 (9%) | 1 (2%) | 0.2 |
| Valvular predisposition  prosthetic valve  other valvular disease | 66 (42%)  44 (28%)  22 (14%) | 17 (41%)  10 (24%)  7 (17%) | 0.9  0.8  0.6 |
| **IE characteristics** |  |  |  |
| Staphylococcus Aureus | 84 (54%) | 10 (24%) | 0.001 |
| Enterococcus sp | 15 (10%) | 2 (5%) | 0.3 |
| Other Streptococci | 34 (22%) | 15 (37%) | 0.06 |
| Other pathogen | 17 (11%) | 11 (26%) | 0.01 |
| Blood culture negative | 6 (4%) | 2 (5%) | 0.7 |
| Prosthetic valve IE | 38 (24%) | 9 (22%) | 0.8 |
| IE topography  Mitral  Aortic  Both | 75 (48%)  61 (39%)  19 (12%) | 14 (34%)  19 (46%)  8 (20%) | 0.1  0.4  0.2 |
| Community acquired | 105 (67%) | 31 (73%) | 0.5 |
| Vegetation >15mm | 69 (44%) | 17 (42%) | 0.7 |
| Severe regurgitation | 64 (41%) | 29 (71%) | 0.001 |
| Cardiac abscess or fistula | 42 (27%) | 11 (27%) | 0.9 |
| Extra-neurological CT-defined embolism | 70 (55%) | 8 (36%) | 0.1 |
| **Baseline clinical characteristics** |  |  |  |
| SOFA | 7 [4-10] | 6 [4-10] | 0.6 |
| “non-neurological” SOFA | 6 [3-9] | 6 [4-8] | 0.8 |
| Mechanical ventilation | 89 (57%) | 25 (61%) | 0.2 |
| IV catecholamines | 65 (42%) | 18 (43%) | 0.9 |
| Lactate>2mmol/L | 40 (26%) | 12 (29%) | 0.6 |
| GCS  GCS<10 | 14 [12-15]  30 (19%) | 15 [13-15]  8 (20%) | 0.4  0.9 |
| Patients referred from another center | 96 (62%) | 28 (67%) | 0.5 |
| **Treatments** |  |  |  |
| Surgical indication identified | 133 (85%) | 37 (90%) | 0.4 |
| No surgery despite indication | 28 (21%) | 8 (22%) | 0.9 |
| Operated | 108 (69%) | 29 (71%) | 0.8 |
| Delay diagnosis-surgery | 7 [4-15] | 4 [2-12] | 0.07 |
| **Outcome** |  |  |  |
| Favorable outcome (mRS 0-3) at one year | 69 (45%) | 15 (37%) | 0.3 |
| Survival at one year | 74 (48%) | 15 (37%) | 0.19 |

1. eGFR<60mL.min^-1^.1.73m^-2^ for >3 months

*IE: infective endocarditis, IV: Intra-venous, CIED: cardiac implantable electronic device, LVEF: left ventricular ejection fraction, CT: computed tomography, SOFA: sequential organ failure assessment, GCS: Glasgow coma scale*
