# Supplementary material for: Cerebrovascular complications and outcomes of critically ill adult patients with infective endocarditis
Source: Ann Intensive Care. 2022 Dec 30;12:119. doi: 10.1186/s13613-022-01086-6 (PMC9803797; doi:10.1186/s13613-022-01086-6)
Supplement: Supplementary file 2 — Additional file 2: Table S2. Univariable analysis of baseline characteristics associated with outcome. [file 13613_2022_1086_MOESM2_ESM.docx]

| **Characteristics** | **Total (n=156)** | **mRS 0-3 at 1 year (n=69)** | **mRS 4-6 at 1 year (n=85)** | **P** |
| --- | --- | --- | --- | --- |
| **Demography** |  |  |  |  |
| Age (years) | 63 [54-70] | 58 [47-67] | 65 [58-72] | 0.00031 |
| Male sex | 110 (71%) | 50 (73%) | 58 (68%) | 0.57 |
| **Pre-existing conditions** |  |  |  |  |
| Charlson score | 1 [0-3] | 0 [0-2] | 1 [0-4] | 0.015 |
| Diabetes | 46 (30%) | 13 (19%) | 33 (39%) | 0.0071 |
| Chronic kidney disease (a) | 30 (19%) | 8 (12%) | 21 (25%) | 0.039 |
| Immunosuppression | 27 (17%) | 12 (17%) | 14 (17%) | 0.90 |
| IV drug use | 7 (5%) | 5 (7%) | 2 (2%) | 0.24 |
| Stroke | 12 (8%) | 2 (3%) | 10 (12%) | 0.067 |
| Anticoagulant treatment | 48 (31%) | 19 (28%) | 29 (34%) | 0.38 |
| Antiplatelet treatment | 34 (22%) | 11 (16%) | 22 (26%) | 0.13 |
| CIED | 14 (9%) | 3 (4%) | 11 (13%) | 0.10 |
| Valvular predisposition  prosthetic valve  other valvular disease | 66 (42%)  44 (28%)  22 (14%) | 28 (40%)  19 (28%)  9 (13%) | 37 (44%)  24 (28%)  13 (15%) | 0.71  0.92  0.69 |
| **IE characteristics** |  |  |  |  |
| Staphylococcus Aureus | 84 (54%) | 34 (49%) | 49 (58%) | 0.30 |
| Enterococcus sp | 15 (10%) | 9 (13%) | 5 (6%) | 0.12 |
| Other Streptococci | 34 (22%) | 15 (22%) | 19 (22%) | 0.92 |
| Other pathogen | 17 (11%) | 9 (13%) | 8 (9%) | 0.47 |
| Blood culture negative | 6 (4%) | 2 (3%) | 4 (5%) | 0.69 |
| Prosthetic valve IE | 38 (24%) | 17 (25%) | 20 (24%) | 0.87 |
| IE topography  Mitral  Aortic  Both | 75 (48%)  61 (39%)  19 (12%) | 28 (41%)  33 (48%)  8 (12%) | 48 (56%)  26 (31%)  11 (13%) | 0.058  0.029  0.80 |
| Community acquired | 105 (67%) | 48 (69%) | 57 (67%) | 0.79 |
| Vegetation >15mm | 69 (44%) | 31 (45%) | 38 (45%) | 0.98 |
| Severe regurgitation | 64 (41%) | 35 (51%) | 28 (33%) | 0.028 |
| Cardiac abscess or fistula | 42 (27%) | 20 (29%) | 22 (26%) | 0.67 |
| LVEF <50% | 64 (41%) | 10 (15%) | 18 (21%) | 0.30 |
| Extra-neurological CT-defined embolism | 70 (55%) | 32 (52%) | 42 (58%) | 0.49 |
| **Baseline clinical characteristics** |  |  |  |  |
| “non-neurological” SOFA | 6 [3-9] | 5 [3-7] | 7 [4-10] | 0.014 |
| Mechanical ventilation | 89 (57%) | 32 (46%) | 56 (66%) | 0.015 |
| IV catecholamines | 65 (42%) | 22 (32%) | 43 (51%) | 0.019 |
| Lactate>2mmol/L | 40 (26%) | 16 (23%) | 24 (28%) | 0.48 |
| GCS  GCS<10 | 14 [12-15]  30 (19%) | 15 [14-15]  7 (10%) | 14 [9-15]  23 (27%) | 0.00091  0.0082 |
| Patients referred from another center  Including referred from another ICU | 96 (62%)  73 (47%) | 43 (62%)  29 (42%) | 53 (62%)  42 (49%) | 0.99  0.36 |

**Additional file Table S2: Univariable analysis of baseline characteristics associated with outcome**

1. eGFR<60mL.min^-1^.1.73m^-2^ for >3 months ^1^

*IE: infective endocarditis, IV: Intra-venous, CIED: cardiac implantable electronic device, LVEF: left ventricular ejection fraction, CT: computed tomography, SOFA: sequential organ failure assessment, GCS: Glasgow coma scale, ICU: intensive care unit*

*As neurological clinical status has already been identified as an independent predictor of outcome, we ought to precisely analyze the independent impact of neuroradiological and neurological clinical data on outcome. We therefore decided to use two separate variables, namely “non neurological SOFA” and “GCS”, rather than SOFA score only in the univariate analysis.*

*Diabetes, CKD and previous stroke are individual components of the Charlson comorbidity index. Therefore, only the later was included in the multivariable analysis.*

*Mechanical ventilation and IV catecholamines are individual components of the non-neurological SOFA. Therefore, only the later was included in the multivariable analysis.*

*Due to collinearity between the presence of a severe regurgitation and the realization of a surgical treatment of IE, the variable “severe regurgitation” was not included in the multivariable analysis.*

1. Chen TK, Knicely DH, Grams ME. Chronic Kidney Disease Diagnosis and Management. JAMA. 2019;322:1294–1304.
