# Supplementary material for: Cerebrovascular complications and outcomes of critically ill adult patients with infective endocarditis
Source: Ann Intensive Care. 2022 Dec 30;12:119. doi: 10.1186/s13613-022-01086-6 (PMC9803797; doi:10.1186/s13613-022-01086-6)
Supplement: Supplementary file 3 — Additional file 3: Table S3. Multivariate analysis of factors associated with favorable outcome (mRS 0-3) in the whole cohort at one year for brain CT categories others than “moderate-to-severe ischemic stroke”. [file 13613_2022_1086_MOESM3_ESM.docx]

Additional file Table S3: multivariate analysis of factors associated with favorable outcome (mRS 0-3) in the whole cohort at one year for brain CT categories others than “moderate to severe ischemic stroke”

1. **Normal brain CT**

| **Variable** | **Univariate OR [95% CI]** | **Univariate p** | **Multivariate OR [95% CI]** | **Multivariate p** |
| --- | --- | --- | --- | --- |
| Normal brain CT | 1.3 [0.7-2.5] | 0.43 | **-** | **-** |
| **Age** | 0.9 [0.9-1.0] | <0.001 | **0.94 [0.92-0.97]** | **<0.001** |
| **GCS score** | 1.2 [1.1-1.4] | <0.001 | **1.24 [1.09-1.42]** | **0.002** |
| Charlson index ≥2 | 0.6 [0.3-1.1] | 0.085 | - | - |
| Non-neurological SOFA≥5 | 0.5 [0.2-0.9] | 0.021 | - | - |
| Mitral valve involvement | 0.5 [0.3-1.0] | 0.05 | - | - |

1. **Minor ischemic stroke**

| **Variable** | **Univariate OR [95% CI]** | **Univariate p** | **Multivariate OR [95% CI]** | **Multivariate p** |
| --- | --- | --- | --- | --- |
| CT defined minor IS | 0.6 [0.3-1.4] | 0.22 | **-** | **-** |
| **Age** | 0.9 [0.9-1.0] | <0.001 | **0.94 [0.92-0.97]** | **<0.001** |
| **GCS score** | 1.2 [1.1-1.4] | <0.001 | **1.24 [1.09-1.42]** | **0.002** |
| Charlson index ≥2 | 0.6 [0.3-1.1] | 0.085 | - | - |
| Non-neurological SOFA≥5 | 0.5 [0.2-0.9] | 0.021 | - | - |
| Mitral valve involvement | 0.5 [0.3-1.0] | 0.05 | - | - |

1. **Cerebral hemorrhage**

| **Variable** | **Univariate OR [95% CI]** | **Univariate p** | **Multivariate OR [95% CI]** | **Multivariate p** |
| --- | --- | --- | --- | --- |
| CT defined cerebral hemorrhage | 1.9 [0.8-4.6] | 0.15 | 2.23 [0.83-6.01] | 0.112 |
| **Age** | 0.9 [0.9-1.0] | <0.001 | **0.95 [0.92-0.97]** | **<0.001** |
| **GCS score** | 1.2 [1.1-1.4] | <0.001 | **1.24 [1.09-1.42]** | **0.002** |
| Charlson index ≥2 | 0.6 [0.3-1.1] | 0.085 | - | - |
| Non-neurological SOFA≥5 | 0.5 [0.2-0.9] | 0.021 | - | - |
| Mitral valve involvement | 0.5 [0.3-1.0] | 0.05 | 0.57 [0.28-1.19] | 0.112 |

*CT : Contrast Tomography, GCS : Glasgow Coma Scale, SOFA : Sequential Organ Failure Assessment*

*A separate regression analysis was performed for each baseline brain CT category. Minor ischemic stroke, cerebral hemorrhage and normal CT were not significantly associated with one year favorable outcome.*
