# Supplementary material for: Cerebrovascular complications and outcomes of critically ill adult patients with infective endocarditis
Source: Ann Intensive Care. 2022 Dec 30;12:119. doi: 10.1186/s13613-022-01086-6 (PMC9803797; doi:10.1186/s13613-022-01086-6)
Supplement: Supplementary file 4 — Additional file 4: Table S4. Sensitivity analysis including the variable “severe regurgitation” of factors associated with favorable functional outcome (mRS 0-3) at one year. [file 13613_2022_1086_MOESM4_ESM.docx]

**Additional file Table S4 sensitivity analysis including the variable “severe regurgitation” of factors associated with favorable functional outcome (mRS 0-3) at one year**

**All patients (n=154)**

| **Variable** | **Univariate OR [95% CI]** | **Univariate p** | **Multivariate OR [95% CI]** | **Multivariate p** |
| --- | --- | --- | --- | --- |
| **CT-defined moderate to severe ischemic stroke** | 0.7 [0.3-1.5] | 0.34 | **0.37 [0.14-0.95]** | **0.038** |
| **Age** | 0.9 [0.9-1.0] | <0.001 | **0.94 [0.91-0.97]** | **<0.001** |
| **GCS score** | 1.2 [1.1-1.4] | <0.001 | **1.23 [1.08-1.42]** | **0.003** |
| Charlson index ≥2 | 0.6 [0.3-1.1] | 0.085 | 0.54 [0.25-1.18] | 0.12 |
| Non-neurological SOFA≥5 | 0.5 [0.2-0.9] | 0.021 | - | - |
| Mitral valve involvement | 0.5 [0.3-1.0] | 0.05 | 0.58 [0.28-1.20] | 0.14 |
| Severe regurgitation | 2.1 [1.1-4.0] | 0.028 | 1.56 [0.75-3.36] | 0.23 |

*CT : Computed Tomography, GCS : Glasgow Coma Scale, SOFA : Sequential Organ Failure Assessment*

*Non-neurological SOFA≥5 was not selected for the final model during the backward selection procedure (p>0.2).*
