# Supplementary figures and images for: Cerebrovascular complications and outcomes of critically ill adult patients with infective endocarditis
Source: Ann Intensive Care. 2022 Dec 30;12:119. doi: 10.1186/s13613-022-01086-6 (PMC9803797; doi:10.1186/s13613-022-01086-6)

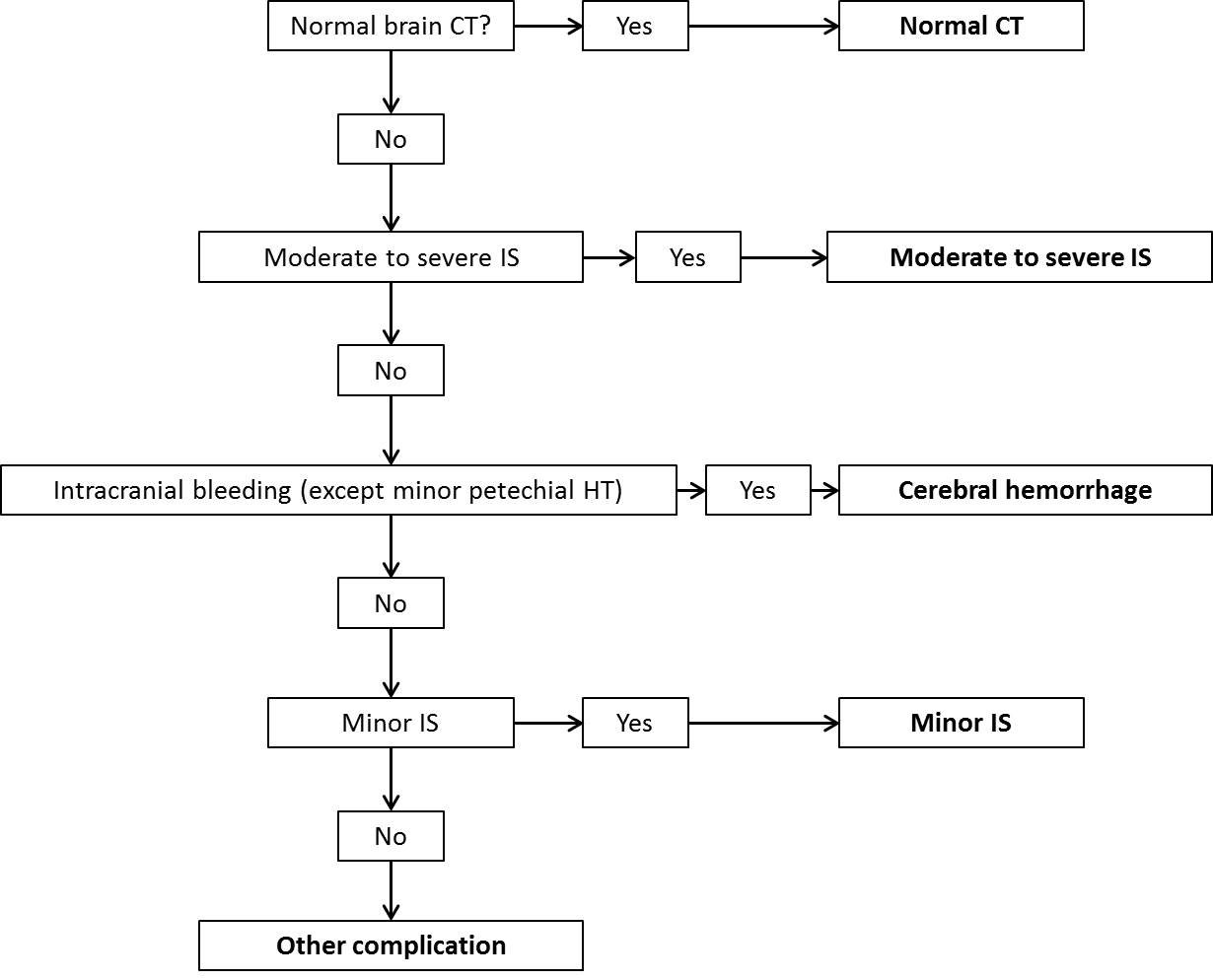

Supplement: Supplementary file 5 — Additional file 5: Figure S1. Details of baseline brain CT classification IS= Ischemic stroke HT=hemorrhagic transformation of an ischemic stroke [file 13613_2022_1086_MOESM5_ESM.jpg]

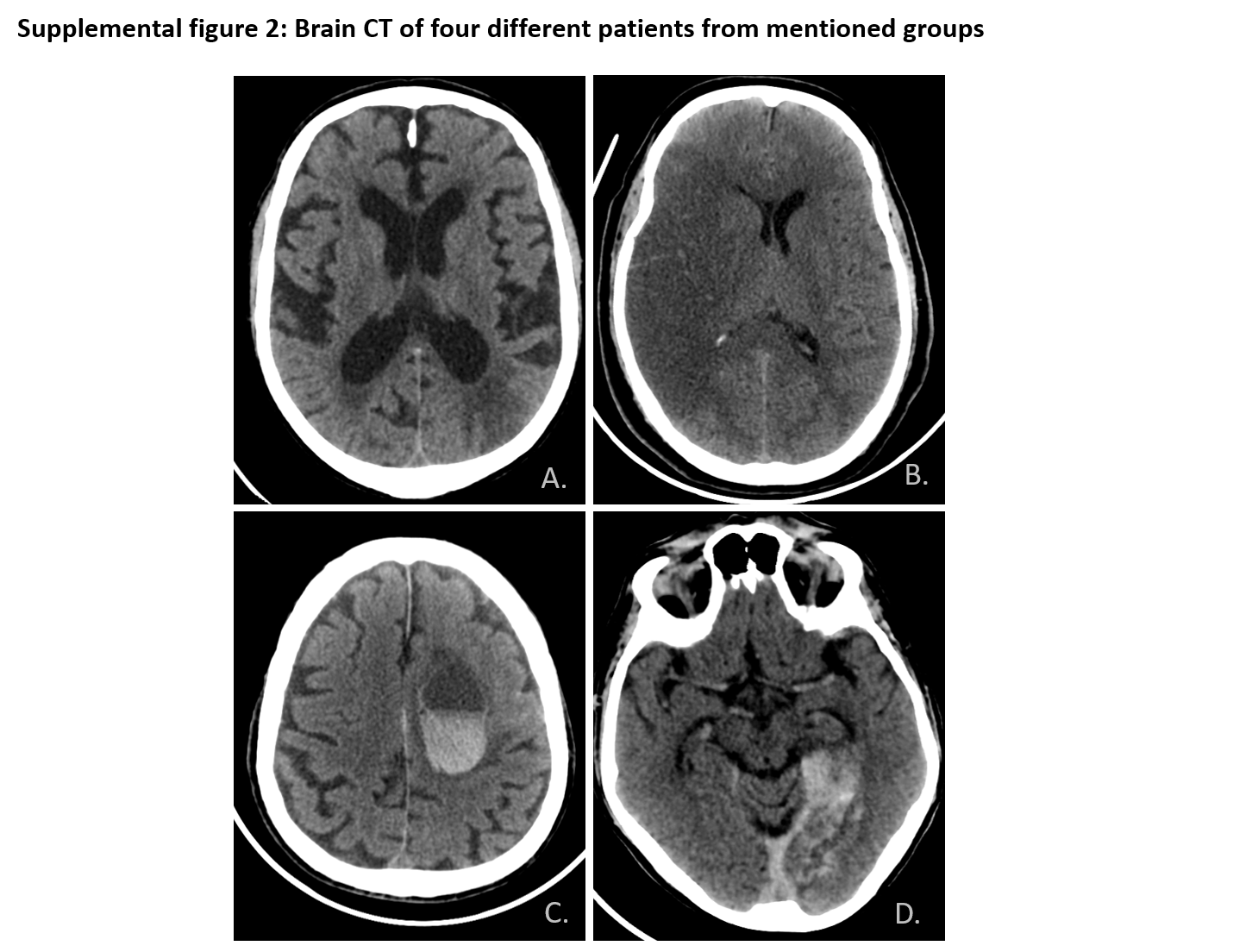

Supplement: Supplementary file 6 — Additional file 6: Figure S2. Brain CT of four different patients from mentioned groups A. minor stroke of a distal branch of the left middle cerebral artery (ASPECTS 9/10, minor ischemic stroke group), B. large stroke involving the right middle cerebral artery with midline shift due to edema and mass effect (ASPECTS 3/10, moderate-to-severe ischemic stroke), C. intraparenchymal in left centrum semiovale with swirl sign (cerebral hemorrhage group) and D. hemorrhagic transformation of a left posterior cerebral artery stroke (also cerebral hemorrhage group). [file 13613_2022_1086_MOESM6_ESM.tif]
